# Pediatric colonic diverticulitis: clinical presentation, management, and review of the literature

**DOI:** 10.1007/s00383-026-06521-4

**Published:** 2026-07-20

**Authors:** M. Pistone, M. Bosisio, L. Wennemann, S. Tohmasi, A. Zani, Elke Zani-Ruttenstock

**Affiliations:** 1 Division of Pediatric Surgery, Department of Surgery, Washington University School of Medicine, 1 Children’s Place, 63110 Saint Louis, MO USA; 2 University of Tor Vergata, Rome, Italy; 3 Department of Clinical and Experimental Sciences, University of Brescia, Brescia, Italy; 4 Department of Pediatric and Adolescent Surgery, Hannover Medical School, Hannover, Germany

**Keywords:** Child, Pediatric, Adolescent, Diverticulitis, Colonic

## Abstract

Colonic diverticulitis is rare in the pediatric population and often presents with nonspecific symptoms that may be misdiagnosed as other causes of acute abdominal pain, such as appendicitis, leading to diagnostic uncertainty and variability in management. We report a case of pediatric colonic diverticulitis and present a systematic review to characterize its clinical presentation, diagnostic challenges and management. Clinical data for the index patient were retrospectively reviewed, and parental consent was obtained. A structured literature search was performed using PubMed, Scopus, and Embase. Studies reporting patients younger than 18 years were included. A 16-year-old male presented with right lower quadrant pain. CT suggested cecal diverticulitis. Worsening symptoms prompted diagnostic laparoscopy, revealing an inflamed diverticulum adjacent to a normal appendix. The postoperative course was uneventful. The review identified 27 studies including 101 patients. Median age was 14 years (IQR 12–16; range 3–17), with 57% male preponderance. Most cases involved a solitary right-sided diverticulum. CT correctly identified diverticulitis in 85%. Complicated disease occurred in 17%. In conclusion, in the pediatric age diverticulitis often arises from a solitary cecal diverticulum and can closely mimic appendicitis. Awareness may improve diagnosis, and uncomplicated cases can be treated conservatively with careful follow-up and appropriate clinical monitoring.

## Introduction

Colonic diverticulitis is a condition typically encountered in adults, with prevalence increasing steadily with age [1]. In contrast, it remains exceedingly rare in the pediatric and adolescent population, with only sporadic case reports and small case series described in the literature. Nevertheless, the number of reported cases appears to be increasing, raising the possibility that this entity is underrecognized rather than truly uncommon [2].

The underlying mechanisms of diverticular disease in younger patients are not fully understood and likely differ from those observed in adults. In adults, diverticular disease is generally considered an acquired condition, associated with increased intraluminal pressure, low fiber diets, and age-related changes in the colonic wall [3]. In children and adolescents, however, diverticular disease may reflect a distinct process, potentially involving congenital or structural factors such as focal weaknesses of the colonic wall or true diverticula.

Despite an extensive body of literature in adults, pediatric colonic diverticulitis remains insufficiently characterized. Its rarity contributes to diagnostic uncertainty and variability in management. This challenge is particularly relevant in the intraoperative setting, where unexpected findings of a normal appendix in the presence of an indeterminate cecal lesion may leave the surgeon without a clear management strategy. Clinical exposure to this entity is limited, even among experienced surgeons, further compounding uncertainty in both diagnosis and treatment [4].

This study aims to systematically compile and analyze all reported cases of pediatric colonic diverticulitis. By providing a structured synthesis of the available data, this study seeks to enhance clinical awareness, support inclusion of this entity in the differential diagnosis of right-sided abdominal pain in children and offer guidance for intraoperative decision making when this condition is encountered unexpectedly.

## Materials and methods

This studycombines a single case from our institution with a systematic review of the literature on colonic diverticulitis in the pediatric population. Clinical data for the index patient were retrospectively obtained from the electronic medical record. Informed parental consent for inclusion and publication was obtained.

**Fig. 1:**
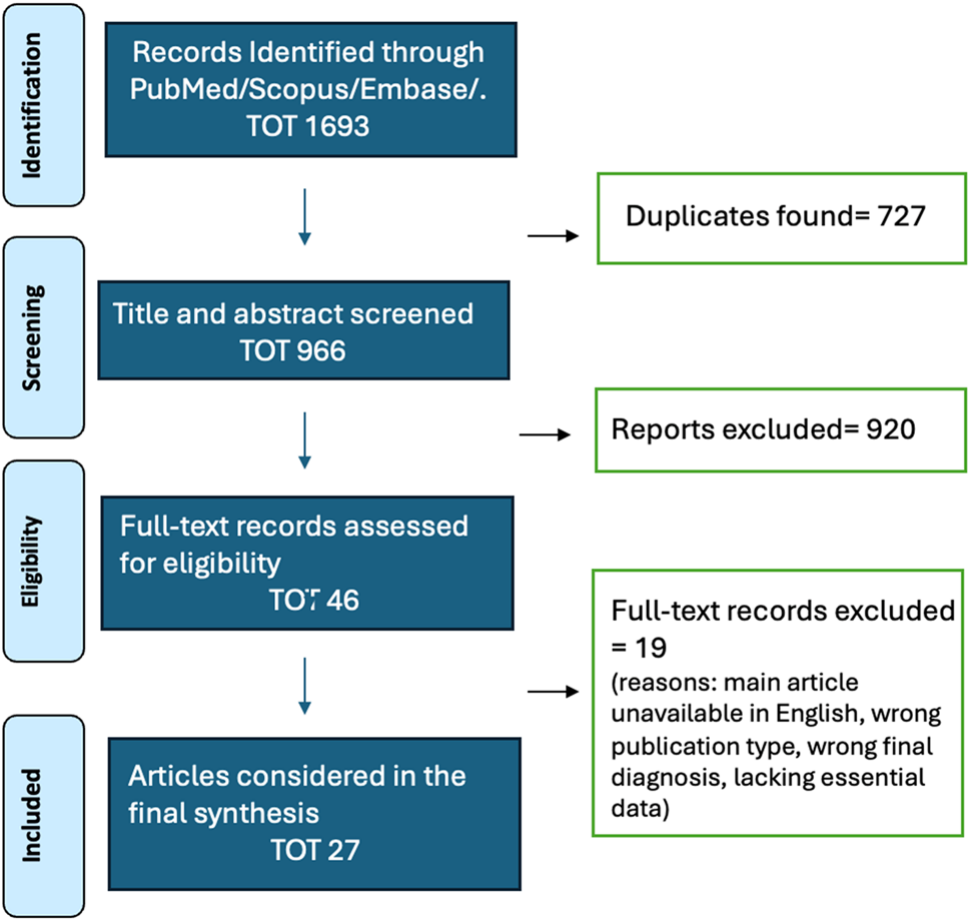
PRISMA flow diagram of study selection process for the literature review

For the systematic review, we followed PRISMA guidelines (Fig. 1) and applied predefined inclusion and exclusion criteria (Table 1). A comprehensive search of PubMed, Scopus, and Embase databases was performed without date restrictions. The search strategy incorporated a combination of the following keywords: “child,” “pediatric,” “adolescent,” “diverticulitis,” and “colonic.”

**Table 1:** Inclusion and exclusion criteria for Systematic Review

Inclusion criteria	Exclusion criteria
Data reported on pediatric patients (< 18) with confirmed colonic diverticulitis.	Article unavailable in English
	Reviews
	Non full articles

Data extraction was performed in a standardized manner and included patient demographics, geographic origin of the study population, associated genetic disorders, presenting symptoms, imaging modality, anatomical location of diverticulitis, number of diverticula (single versus multiple), and diagnostic accuracy both, preoperatively and intraoperatively. Additional variables included complication rates (perforation, fistula formation, and abscess) and histopathologic classification (true versus false diverticula).

To account for potential differences arising from varying levels of clinical experience with this condition, additional subanalyses were performed in selected instances. When appropriate, analyses were repeated with exclusion of the two largest case series to evaluate the robustness of findings independent of cohorts with greater familiarity and experience with this disease.

As 18 years is the conventional cutoff for pediatric care and typically defines presentation to pediatric surgical services, only studies reporting data specific to patients younger than 18 years were included. Only one study, the largest identified case series, reported a mixed cohort including patients up to 20 years of age. The corresponding author of this study was therefore contacted to obtain age-specific data.

In addition to the cases identified through the systematic review, the index case from our institution was included in the summary table for descriptive purposes only and was not part of the systematic analysis.

## Results

### Institutional case

**Fig. 2:**
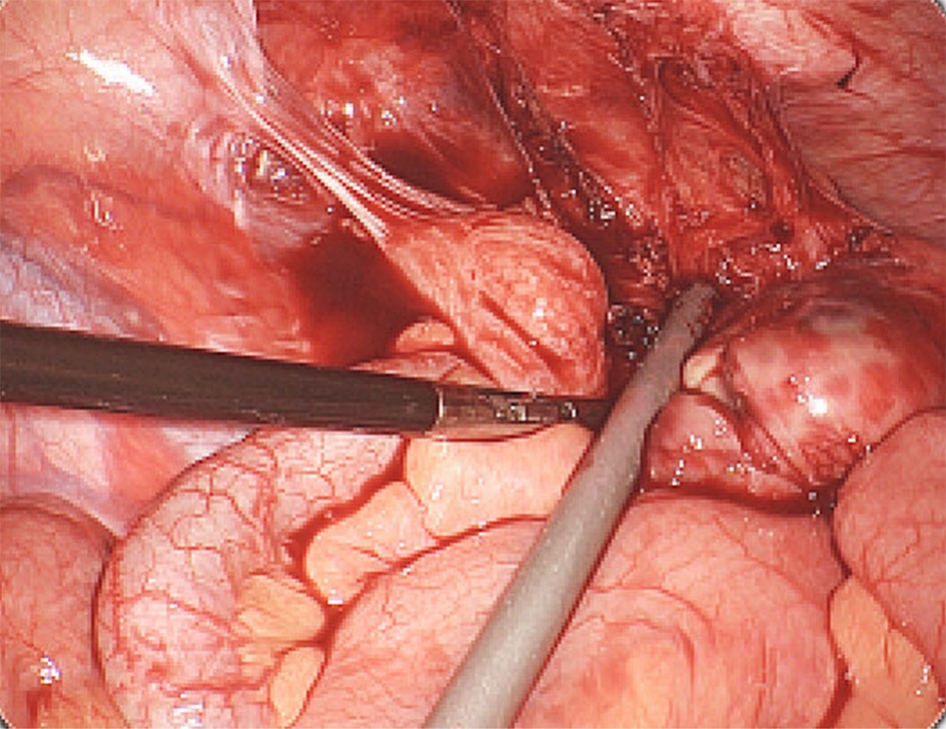
Intraoperative view at the inflamed cecal diverticulum ascending from the anterior aspect of the cecal wall and with its tip strongly adherent to the parietal peritoneum of the right lower quadrant adjacent to normal appendix and normal terminal ileum

**Fig. 3:**
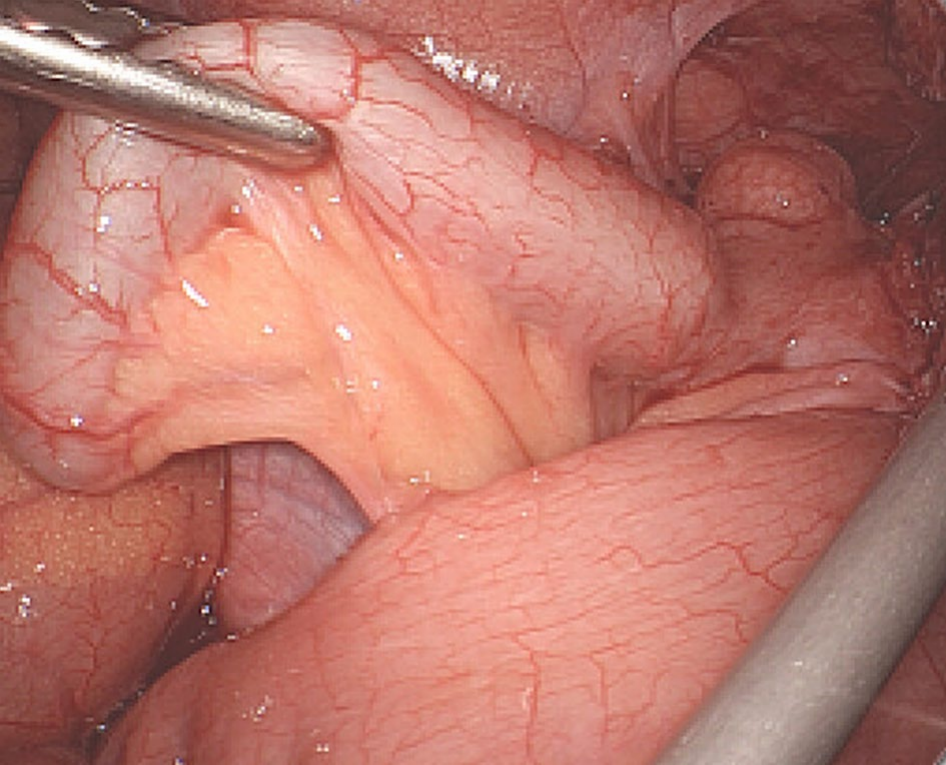
Intraoperative view of the normal appendix and normal terminal ileum in the right lower quadrant

A 16-year-old male of Asian descent, with no significant past medical history, presented with same day onset of right lower quadrant abdominal pain without associated fever or other systemic symptoms. He was initially evaluated at an outside hospital, where abdominal ultrasound was inconclusive, and a subsequent computer tomography (CT) scan raised concern for right sided colonic diverticulitis. Based on these findings, he was transferred to our institution for further management. On arrival at our emergency department, the patient reported near complete resolution of pain. Physical examination was benign, without signs of peritonitis, and laboratory studies showed mild leukocytosis (WBC 12 × 10^9^/L) and an elevated C reactive protein (54 mg/L). In the setting of clinical stability, the patient was discharged home on oral amoxicillin-clavulanate with close outpatient follow-up. He returned the following day with recurrent and worsening right lower quadrant pain and new onset anorexia. Given the evolution of symptoms and ongoing diagnostic uncertainty, operative exploration was pursued. Diagnostic laparoscopy revealed an inflamed diverticulum arising from the anterior aspect of the cecum (Fig. 2) without signs of perforation. After careful mobilization, the appendix was identified and appeared macroscopically normal (Fig. 3). Appendectomy was deferred given the normal appearance of the appendix and the concern that resection and stump healing could delay subsequent endoscopic evaluation. The postoperative course was uneventful. The patient was discharged on postoperative day 1 with a 7-day course of oral ciprofloxacin and metronidazole and advised to follow a low residue diet. Outpatients follow up with gastroenterology was arranged, with colonoscopy recommended during a quiescent phase. At the time of writing, the patient has been followed for approximately 4.5 months and remains asymptomatic without evidence of recurrence. Surgical resection was considered in the event of recurrent or complicated diverticulitis.

### Systematic review

A total of 27 studies met inclusion criteria, comprising 24 case reports [5–28] and 3 case series [2, 4, 29], yielding a total of 101 patients (Table 2). In the largest included case series, age-restricted data for patients younger than 18 years (*n* = 53) were directly obtained from the authors and incorporated into the analysis. The median age at presentation was 14 years (IQR 12–16; range 3–17), with 57% male preponderance. Most cases (77%) originated from the Asian population.

**Table 2:** Case reports and case series of colonic diverticulitis in pediatric patients

Year	Author	# Of cases	Sex	Age	Country of origin	Genetic disorder	Imaging modality reported	Location	Single/ Multiple	Initial (PreoperaTIVE) diagnosIs	Intraoperative diagnosIs (when surgery performed)	Complications	Histology (True/False Diverticulum)
*Case reports*													
1975	Barlow et al. [5]	1	F	8	USA	No	Us, ct	Cecum	Single	Appendicitis	Double appendix appendicitis	Perforation	Nd
1977	Rees et al. [6]	1	M	9	USA	No	Enema	Sigmoid	Multiple	Diverticulitis	Diverticulitis	Rectovescical fistula	Nd
1983	Halata et al. [7]	1	M	16	USA	No	US, enema, upper GI	Sigmoid	Multiple	Diverticuitis/mass	Diverticulitis	Abscess	Nd
1988	Wilkinson et al. [8]	1	F	13	Australia	No	Us	Right/transverse colon	Single	Appendicitis	Appendicitis	Abscess	True
1997	Benya et al. [9]	1	F	13	USA	CF	Us	Right colon	Single	Mass	Mass	No	False
2005	Sigaloff et al. [10]	1	M	13	NL	No	Us	Cecum	Single	Appendicitis	Mass	Perforation	False
2005	Deshpande et al. [11]	1	M	17	Australia	WS	Ct	Sigmoid	Multiple	Diverticulitis/ischemic colitis	Diverticulitis	Perforation and abscess	True
2009	Garcia et al. [12]	1	M	13	USA	WS	Ct	Sigmoid	Multiple	Appendicitis	Diverticuliis	Colovesicular and Coloenteric fistulae	Nd
2009	Santin et al. [13]	1	M	15	USA	WS	Ct	Sigmoid	Multiple	Diverticulitis	Diverticulitis	No	Nd
2011	Cheng et al. [14]	2	2 f	3; 15	USA	No	2 Ct	2 cecum	2 single	2 appendicitis	Mass; diverticulitis	Perforation x2, abscess	False x2
2012	Ignacio et al. [15]	1	M	9	USA	WS	Ct	Sigmoid	Single	Polyps/inflammatory disease	Mass	No	Nd
2016	Hungtington et al. [16]	1	M	9	USA	No	Us, ct	Cecum	Single	Appendicitis	Mass	No	True
2016	Ho et al. [17]	1	F	12	Taiwan	No	Us, ct	Cecum	Multiple	Diverticulitis	Mass	No	Nd
2017	Horton et al. [18]	1	M	13	USA	No	Us, ct	Cecum	Multiple	Mass	Mass	No	Nd
2018	Setlur A et al. [19]	1	M	17	USA	No	Ct	Sigmoid	Unspecified	Diverticulitis	–	Perforation, abscess	Nd
2019	Çil et al. [20]	1	M	14	Turkey	No	Us	Cecum	Single	Appendicitis	Diverticulitis	Perforation	Nd
2019	Spacil et al. [21]	1	F	15	Germany	No	Us	Cecum	Single	Appendicitis	Diverticulitis	Perforation	Nd
2021	Bartel et al. [22]	1	M	15	USA	No	Ct	Sigmoid	Single	Diverticulitis	–	No	Nd
2021	Abuelazm S et al. 23]	1	M	16	USA	No	Ct	Cecum	Single	Appendicitis	Diverticulitis	Perforation	Nd
2021	Lee et al. [24]	1	F	11	USA	No	Us, ct	Cecum	Single	Diverticulitis	–	No	Nd
2022	Anadolulu Aİ et al. [25]	2	M; f	8 × 2	Turkey	No	Ct	2 cecum	2 single	Diverticulitis Appendicitis	Diverticulitis Mass	No	Nd
2025	Fujii T et al. [26]	1	M	15	Japan	No	Us, ct	Right colon	Single	Diverticulitis	–	No	Nd
2025	Özcan H et al. [27]	1	F	15	Turkey	No	Us	Cecum	Single	Appendicitis	Diverticulitis	No	Nd
2025	Aziz HM et al. [28]	1	F	16	Saudi Arabia	No	Ct	Sigmoid	Multiple	Diverticulitis	Diverticulitis	Colovoescicular and utero-vescicular fistulas	Nd
2026	Pistone et al.	1	M	16	USA	No	Us,** ct**	Right colon	Single	Diverticulitis/ Appendicitis	Diverticulitis	No	Nd
*Case series*													
2021	Hatakeyama et al. [4]	16	5 m, 11 f	12 (8–15)	Japan	No	14 us 3 ct	All cecum	16 single	16 diverticulitis	–	No	Nd
2024	Han et al. [2]	53	33 m, 20 f	16 (7–17)	South Korea	No	6 us 53 ct	All right colon	47 single 6 multiple	49 diverticulitis 4diverticulitis/ Appendicitis	4 diverticulitis	No	Nd
2024	Li et al. [29]	6	4 m 2 f	10.5 (4–12)	China	No	6 us	All cecum	6 single	3 diverticulitis 3 appendicitis	3 missed diagnosis, unspecified	Perforation X3	Nd

*CF* Cystic Fibrosis, *WS* Williams Syndrome

Genetic disorders were reported in 5 patients, including 4 with Williams syndrome, a neurodevelopmental disorder characterized by connective tissue abnormalities, and 1 with cystic fibrosis.

Clinical presentation varied in duration from less than one day to several months. Abdominal pain was universal (100%), followed by fever (32%), nausea or vomiting (19%), anorexia (15%), diarrhea (5%) and constipation (4%).

Ultrasound findings were reported in 40 patients (40%) and correctly identified colonic diverticulitis in 45% of cases. CT imaging was performed in 74 patients (73%), and demonstrated higher diagnostic accuracy, correctly identifying diverticulitis in 85%. When excluding the two largest case series by *Han* et al. [2] and *Hatakeyama* et al. [4] the diagnostic accuracy of CT decreased to 56%.

Anatomically, the ascending colon, predominantly the cecum, was involved in 91% of cases. A solitary diverticulum was identified in 87% of patients. In contrast, sigmoid involvement was uncommon (9%), including six cases with multiple diverticula, two with solitary diverticula, and one unspecified.

Overall, colonic diverticulitis was considered in the initial differential diagnosis in 83 patients (82%). Among those who were preoperatively misdiagnosed (23 patients; 23%), 19 (86%) underwent diagnostic laparoscopy for presumed appendicitis, while others were explored for suspected cecal mass, inflammatory disease, polyps, or volvulus. Excluding the two largest case series by *Han* et al. [2] and *Hatakeyama* et al. [4], which reported 100% diagnostic accuracy, the rate of correctly identified cases decreased to 26%.

Overall, 28 patients (28%) underwent surgical management. In 8 cases (29%), diverticulitis was not recognized intraoperatively and was only identified on subsequent histopathologic evaluation. Seventeen patients (17%) presented with complicated disease. The most frequent complications were perforation (*n* = 12), followed by abscess formation (*n* = 4) and fistulas (*n* = 3). When excluding the two largest case series, which predominantly included conservatively managed patients, the rate of complicated disease increased to 53%.

Histopathologic data were available in 10 cases; among the eight cases specifying diverticulum type, there was an equal distribution between true and false diverticula. Notably, all reported false diverticula originated from the right colon.

## Discussion

The present study provides a comprehensive synthesis of all reported cases of colonic diverticulitis in the pediatric population to date, regardless of management strategy. By integrating both, medically and surgically managed cases, this analysis offers a clinically relevant overview of a rare entity that is often encountered unexpectedly, particularly in the intraoperative setting.

From a demographic perspective, pediatric colonic diverticulitis most commonly affects adolescents, with a median age of 14 years and a slight male predominance. A striking observation is the predominance of cases originating from Asian populations. While this may reflect true geographic variation, it is likely also influenced by increased clinical familiarity and recognition in regions where the condition appears more frequently [30, 31]. This is further supported by the higher diagnostic accuracy and greater use of non-operative management reported in the largest Asian case series [2, 4].

Genetic and syndromic conditions were infrequently reported in our cohort, with only 5 patients affected, including 4 with Williams syndrome and one patient with cystic fibrosis. Williams syndrome is associated with connective tissue abnormalities, which may predispose to structural weakness of the colonic wall and diverticulum formation. Interestingly, other connective tissue disorders that have been implicated in early onset diverticular disease in adults, such as Marfan syndrome and Ehlers-Danlos syndrome, were not reported in the pediatric cases included in this analysis [13]. This may reflect the rarity of these conditions, underreporting in the literature, or potential differences in the pathophysiology of diverticular disease in children compared to adults.

Anatomically, the disease differs fundamentally from the adult pattern. In contrast to the sigmoid predominance seen in Western adults [32], pediatric cases are overwhelmingly right sided, most commonly involving a solitary cecal diverticulum. This pattern appears consistent across geographic regions, suggesting that right-sided, solitary diverticulitis represents the typical presentation in adolescents, regardless of country of origin [14, 16, 23, 25, 27]. The predominance of solitary lesions has been proposed to support a distinct pathophysiological entity, potentially related to congenital or structural factors. However, histopathologic data in our cohort do not fully support this assumption. Among the limited number of cases with available histological analysis (*n* = 8), an equal distribution of true and false diverticula was observed, arguing against the notion that right sided diverticula in this population are uniformly congenital.

The clinical presentation closely mimics that of acute appendicitis, with abdominal pain being universal and frequently localized to the right lower quadrant. Given this overlap, it is not surprising that cecal diverticulitis is frequently misdiagnosed preoperatively. In our cohort, preoperative diagnostic accuracy was limited, particularly when excluding high-volume series with greater disease familiarity. This highlights a key clinical challenge: unless specifically considered, cecal diverticulitis is unlikely to be identified prior to surgery.

Imaging plays a critical role in improving diagnostic accuracy yet remains imperfect. Ultrasound demonstrated a diagnostic accuracy of 45%, likely reflecting its operator dependence and limited familiarity with this condition in pediatric practice. CT scan performed better, correctly identifying diverticulitis in 85% of cases. However, after excluding the two largest series, even CT failed to establish the diagnosis in a substantial proportion of patients. Importantly, when diverticulitis is correctly identified preoperatively, non-operative management is often feasible, particularly in uncomplicated cases without perforation, abscess, or fistula. These findings underscore the importance of maintaining a low threshold for advanced imaging when clinical features are atypical for appendicitis.

The intraoperative setting represents a second critical diagnostic juncture. Even when surgery is undertaken for presumed appendicitis, diverticulitis may not be immediately recognized. In our analysis, intraoperative identification occurred in only 71% of cases with an incorrect preoperative diagnosis. Distinguishing a cecal diverticulum from other pathologies, such as inflammatory bowel disease or neoplastic lesions, can be challenging, and uncertainty may lead to overtreatment, including unnecessary ileocecal resection.

Our institutional case illustrates this dilemma. Despite preoperative imaging raising suspicion for diverticulitis, the diagnosis remained uncertain, reflecting the limited clinical exposure to this condition, even among experienced surgeons. Diagnostic laparoscopy proved to be a valuable tool, allowing confirmation of a non-perforated cecal diverticulum in the presence of a normal appendix. In the absence of complications, a conservative surgical approach was adopted, deferring appendectomy to avoid potential complications related to stump healing and to facilitate timely postoperative endoscopic evaluation. This approach aligns with the broader observation that uncomplicated pediatric diverticulitis can often be managed without resection.

Notably, differences between case reports and larger series further highlight the impact of clinical experience. While individual case reports frequently describe unexpected intraoperative findings and operative management, the two largest case series demonstrate higher diagnostic accuracy, along with a greater reliance on conservative treatment strategies [2, 4]. This suggests that increased familiarity with the condition directly influences both diagnostic confidence and management decisions.

Several practical implications arise from these findings. Although rare, pediatric colonic diverticulitis should be considered in the differential diagnosis of right sided abdominal pain, particularly when imaging findings are atypical for appendicitis or when the appendix appears normal. A low threshold for diagnostic laparoscopy is appropriate in cases of diagnostic uncertainty. However, in the absence of perforation, abscess, or fistula, restraint should be exercised with respect to bowel resection, as unnecessary ileocecal resection may be avoided. Furthermore, collaboration and knowledge exchange with centers that have greater experience with this condition may be valuable in improving recognition and management.

This study has several limitations. The rarity of the condition and the reliance on case reports and small series introduce heterogeneity and potential publication bias, with a likely overrepresentation of more complex or surgically managed cases. In addition, variability in diagnostic approaches across studies limits the ability to draw definitive conclusions regarding optimal imaging strategies or true incidence.

In conclusion, pediatric colonic diverticulitis is a rare but clinically relevant condition that remains underrecognized. Its presentation frequently mimics appendicitis, and both preoperative and intraoperative diagnosis can be challenging. Increased awareness of its characteristic features—particularly right sided, solitary cecal disease in adolescents—may improve diagnostic accuracy and guide more appropriate, often conservative, management. For the pediatric surgeon, familiarity with this entity is essential, particularly when encountered unexpectedly in the operating room.

## Data Availability

All data supporting the findings of this study are available within the paper.
